# Tramiprosate, a drug of potential interest for the treatment of Alzheimer's disease, promotes an abnormal aggregation of tau

**DOI:** 10.1186/1750-1326-2-17

**Published:** 2007-09-06

**Authors:** Ismael Santa-Maria, Félix Hernández, Joaquín Del Rio, Francisco J Moreno, Jesús Avila

**Affiliations:** 1 Centro de Biología Molecular "Severo Ochoa" CSIC/UAM, Fac. Ciencias, Universidad Autónoma de Madrid, Cantoblanco, 28049 Madrid, Spain; 2 Division of Neurosciences, CIMA, School of Medicine, University of Navarra, Av. PioXII, 55, 31008 Pamplona, Spain; 3 CIBERNED, Spain

## Abstract

Alzheimer's disease (AD) is characterized by the presence of two histopathological hallmarks; the senile plaques, or extracellular deposits mainly composed of amyloid-β peptide (Aβ), and the neurofibrillary tangles, or intraneuronal inclusions composed of hyperphosphorylated tau protein.

Since Aβ aggregates are found in the pathological cases, several strategies are under way to develop drugs that interact with Aβ to reduce its assembly. One of them is 3-amino-1-propane sulfonic acid (Tramiprosate, 3-APS, Alzhemed™), that was developed as a sulfated glycosaminoglycan mimetic, that could interact with Aβ peptide, preventing its aggregation.

However, little is known about the action of 3-APS on tau protein aggregation. In this work, we have tested the action of 3-APS on cell viability, microtubule network, actin organization and tau aggregation. Our results indicate that 3-APS favours tau aggregation, in tau transfected non-neuronal cells, and in neuronal cells. We also found that 3-APS does not affect the binding of tau to microtubules but may prevent the formation of tau-actin aggregates. We like to emphasize the importance of testing on both types of pathology (amyloid and tau) the potential drugs to be used for AD treatment.

## Background

Alzheimer's disease is characterized by the presence of two histopathological hallmarks; the senile plaques or extracellular deposits mainly composed of amyloid-β peptide (Aβ) and the neurofibrillary tangles or intraneuronal inclusions composed of hyperphosphorylated tau protein [1].

It has been proposed that some compounds like sulfated glycosaminoglycans (sGAG) [2] could promote the aggregation of Aβ and tau [3-5], and it has been even suggested that in Alzheimer's disease sGAG may provide a common link for Aβ and tau polymerization [3-8].

sGAG including heparan, keratan and chondroitin sulfates strongly favor Aβ polymerization *in vitro *[5,8,9]. Different sGAG also facilitate the assembly *in vitro *of tau [3,4,10]. In addition Aβ [7,11] and tau aggregates [12] associate to sGAG in vivo. On the other hand, the binding of sGAG to Aβ has been found to decrease Aβ degradation [13]. The interaction of the sulfated GAG appear to be through basic residues of the interacting proteins like Aβ [14], supporting previous studies demonstrating the importance of the sulfate moeities of sGAG for the formation of amyloid fibrils [15].

Since Aβ aggregates are found in Alzheimer's disease, several strategies to develop drugs that interact with Aβ to reduce its assembly are under way. Aβ peptide has been taken as a suitable target to develop a therapy against Alzheimer's disease as, at present, the prevalent theory of Alzheimer's disease pathophysiology, the amyloid cascade, hypothesizes that a reduction of Aβ may not only improve amyloid pathology, but also tau pathology [16]. In this way, the action of some compounds have been only tested on amyloid but not on tau pathology [17,18]. One of these compounds is 3-amino-1-propane sulfonic acid (tramiprosate, 3-APS), also known as Alzhemed™ (the use of its trade name is only for identification purposes) [19,20], that was developed as a sGAG mimetic [21,22] that could interact with Aβ peptide, preventing its aggregation.

Not only the contribution of the different forms of Aβ to Alzheimer's disease pathology is uncertain and recent evidence rather implicates soluble oligomers [23], but it is generally accepted that blocking tau pathology could have therapeutic benefit. In this work, we have tested the action of 3-APS on tau aggregation. Our results indicate that 3-APS favours tau aggregation, but with a different mechanism to that found for other tau assembly inducers, like heparin. We also found that 3-APS does not affect the binding of tau to microtubules but may prevent the formation of tau-actin aggregates.

## Results

### Effect of 3-APS on tau stably transfected HEK 293 cells

**Figure 1:**
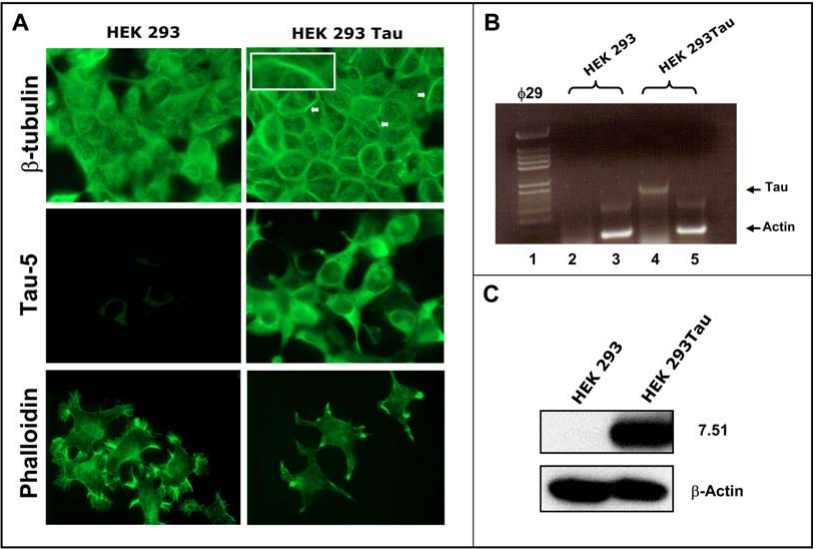
**Levels of Tau in HEK 293 expressing Tau cell line**. A) Immunofluorescence analysis of both HEK 293 and HEK 293 expressing Tau cell lines using antibodies against β-tubulin, tau protein (Tau-5) and phalloidin to identifiy actin polymers. The results obtained for untransfected (left) and tau-transfected (right) cells are shown. In the presence of Tau, tubulin bundles were observed (see arrows). Inset shows a microtubule bundle inside HEK 293 tau expressing cells. B) RNA was isolated from previously mentioned cell lines as described in Materials and methods and a quantitative RT/PCR analysis using actin RNA, as internal control, was done. The size of the amplified DNA was determined by gel electrophoresis; Tau DNA, lanes 2 and 4 and actin DNA (lanes 3 and 5) are shown. In lane 1, are Hind III fragments of φ29 DNA used as electrophoretic markers. The quantitation of the ratio Tau/actin in arbitrary units is shown. C) Total protein extract from HEK 293 and HEK 293 expressing tau was obtained and Western blot analysis using an antibody against β-actin and an antibody (ab 7.51) against Tau, was done.

To study the direct effect of 3-APS on tau protein without the interference of other neuronal proteins, non neuronal cells, HEK 293, lacking tau were stably transfected with human tau cDNA. The tau-expressing cells were identified by immunofluorescence using an antibody raised against tau. Figure 1 indicates that upon tau expression the transfected cells show some microtubules bundles (see arrows and inset in Figure 1A), and that the actin-stress fibers dissappear. Moreover, some aggregates, where tau and actin colocalize, were found at the cell membrane (see below). These aggregates could be similar to those recently reported in Drosophila cells [24]. In addition, the presence and quantitation of tau mRNA by RT-PCR, (Figure 1B) was determined, and the expression of tau protein was also analyzed by Western blot (Figure 1C).

Quantitation of tau protein expressed in these cells, indicated that tau was about 0.1 % of the total soluble cell protein. Since the content of tubulin in these cells was about 2 %, the ratio tau/tubulin was in line to that described for neuronal cells [25].

**Figure 2:**
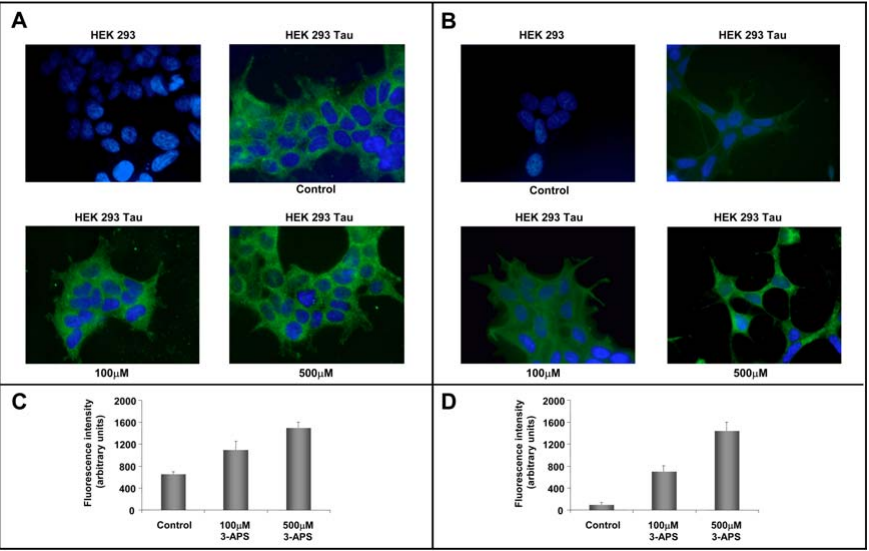
**Effect of 3-APS on Tau transfected cell line**. HEK 293 expressing Tau cells were treated with 100 μM and 500 μM of 3-APS and stained using an antibody against human Tau (ab T14) (A) or with 0.01 % thioflavin S (Th-S) (B). Quantitation of both T14 (C) and Thioflavin S (D) fluorescence observed by fluorescence microscopy is shown. The fluorescence intensity of each sample was obtained by background subtraction. A significant fluorescence intesity increase (P < 0.05 as compared with untreated cells) is shown for both T14 and Thioflavin S staining. The average of at least three separate determinations is indicated. DAPI was used for nuclei staining.

Increasing amounts of 3-APS were added to untransfected or transfected HEK 293 cells. Figure 2 indicates that upon addition of increasing amounts of 3-APS, a slight increase in tau staining was observed in tau transfected cells (see Figures 2A, 2C). This was not due to an increase in the amount of tau, as determined by Western blot (not shown), but probably, to an increase in tau aggregation in treated cells. Since tau aggregates are usually stained with Thioflavin S (Th-S) [26], Th-S staining was performed on the transfected cells after adding increasing amounts of 3-APS. Figure 2B also shows that there was a clear increase in Th-S staining dependent upon addition of increasing amounts of 3-APS (see also Figure 2D).

**Figure 3:**
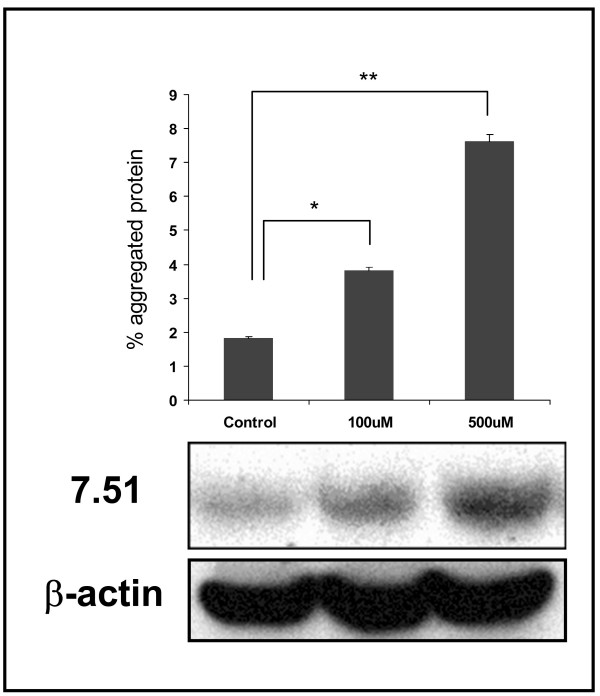
**Tau aggregation in HEK 293 Tau expressing cells treated with 3-APS**. Detergent insoluble aggregates of Tau were isolated from HEK 293 tau cells untreated (control) or treated with 3-APS. The detergent insoluble material (see methods) was analyzed by western blot using an antibody against Tau (ab 7.51). Reaction of the unfractionated cell extract with β-actin antibody, was used as loading control. The average of at least three separate determinations is indicated. *, P < 0.05, **, P < 0.01 compared with control (Student's t test).

To confirm the increase in tau aggregates in 3-APS-treated cells, detergent insoluble aggregates were isolated and the presence of tau protein in those aggregates was determined. Western blot analysis revealed the presence of tau in the detergent insoluble fraction, and also showed that the amount of tau in the aggregates increased with the concentration of added 3-APS (Figure 3). When the same experiments, shown in Figures 2 and 3, were done in the presence of a sGAG, like heparin, instead of 3-APS, no differences were found with respect to controls (absence of heparin or 3-APS), when tau aggregation was tested. It can be explained by the fact that 3-APS, but not heparin, can enter into the cell.

### 3-APS facilitates tau assembly *in vitro*

**Figure 4:**
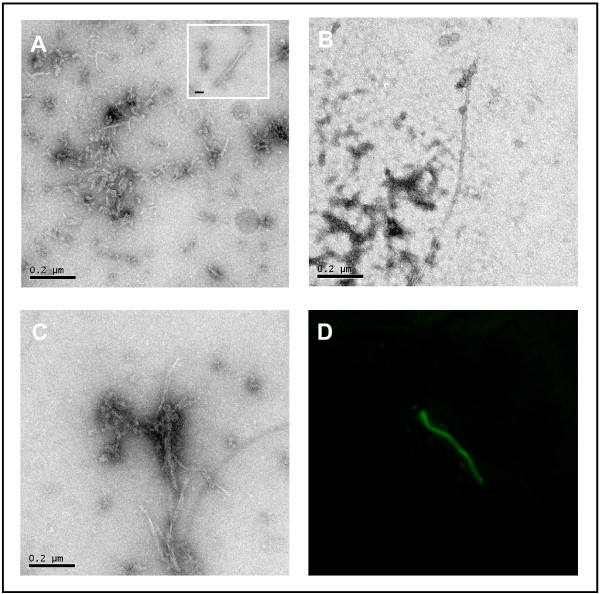
**Polymerization of Tau in the presence of 3-APS**. Fibrillar Tau polymers assembled in the presence of 3-APS were observed by Electron Microscopy. Mainly, short fibrillar and occasionally longer and wider filaments (inset) were found (A). These polymers are not identical to those found in the presence of heparin (B). An increase in both types was found in the presence of both heparin and 3-APS (C). Bars indicate 0.2 μm. 3-APS induced tau polymerization stained with Th-S and visualized by immunofluorescence (see methods) (D).

To test if 3-APS directly facilitated the assembly of tau protein, purified recombinant tau protein was mixed with 3-APS and, after incubation of the mixture (in the conditions described in Materials and Methods), the aggregates were visualized under electron microscopy. Figure 4A shows the presence of short fibrillar polymers, upon addition of 3-APS; together with fewer but longer and larger fibrillar polymers (inset of the figure). No polymers were found in the absence of added tau. Some of the longer and larger 3-APS induced polymers were similar to those assembled in the presence of heparin (Figure 4B). When tau protein was mixed with both 3-APS and heparin both types of polymers were observed (Figure 4C). In addition, we studied the formation of these aggregates by immunofluorescence analysis [27], and we found that they could be identified by Th-S staining (Figure 4D). The result shown in Figure 4D suggests that the increased Th-S staining found upon 3-APS addition could be due to the presence of tau aggregates.

### 3-APS is not affecting to microtubule network, but it could affect to tau-actin interaction

The previous observation suggests that 3-APS binds to tau. This binding could affect the interaction of tau with microtubules and could result in a disorganization of microtubule network. To test this possibility, immunofluorescence analyses to visualize tubulin and tau were carried out. No main differences were found upon addition of 3-APS in microtubule network. Moreover, the *in vitro *binding of tau to microtubules was studied in the absence or presence of 3-APS. Our results indicated that in the presence of 0.5 mM 3-APS there is a slight decrease of *in vitro *tau binding to microtubules (Additional File 1).

**Figure 5:**
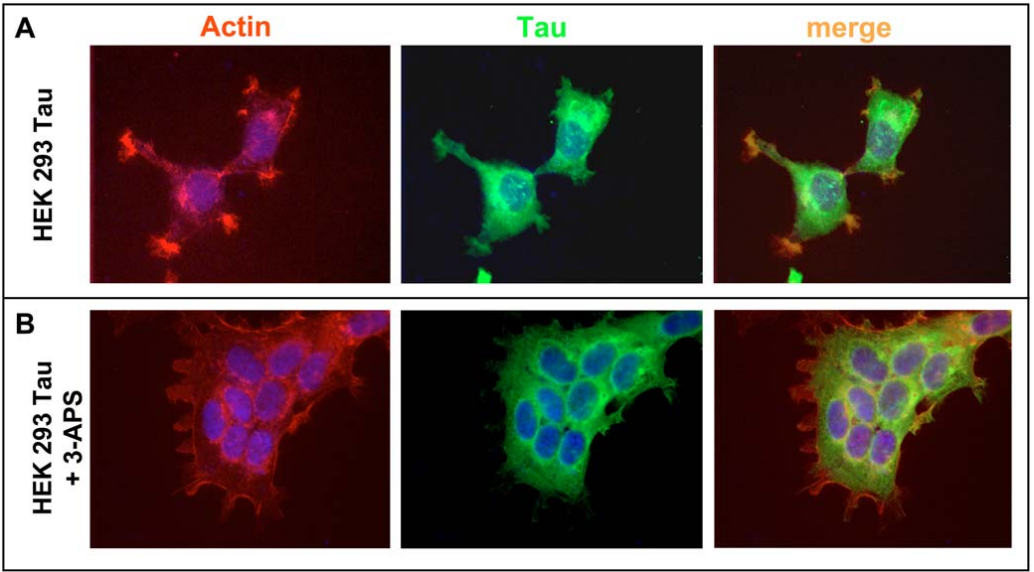
**Effect of 3-APS on tau-actin complexes**. As indicated in Figure 1, tau expresing HEK cells show some lamellipodia regions that are stained by both, phalloidin (aggregated actin) and tau antibodies. The merged picture is also indicated. DAPI was used for nuclei staining (A). In the presence of 200 μM 3-APS, the phalloidin and Tau staining clearly decreases (B).

Although the presence of 3-APS does not affect to the organization of microtubule cytoskeleton, it seems to alter some aspects related to tau-actin aggregates. In Figure 1, it was shown that tau expression in HEK 293 cells resulted, in keeping with previously reported data [28], in the loss of actin stress fibers and in the presence of some aggregates in lamellipodia-like structures that are stained with phalloidin (aggregated actin) and tau antibodies (recently, in PC12 cells, tau has been localized to lamellipodia-like structures, where it associates with actin [29]). These aggregates are shown in Figure 5A. Upon addition of 3-APS these aggregates disappeared (Figure 5B) suggesting that addition of 3-APS could result in the disasembly of tau-actin aggregates.

### 3-APS is bound to tau when the C-terminal region of the protein is present

**Figure 6:**
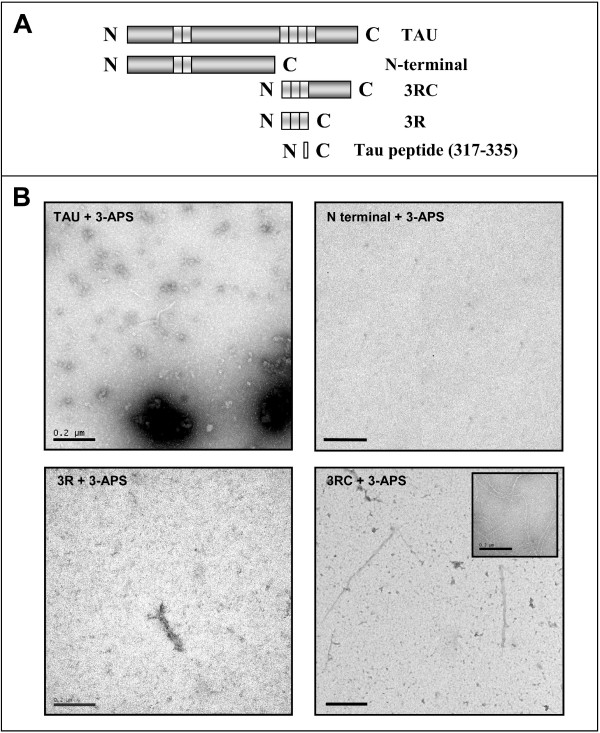
**Polymers found when 3-APS was added to tau protein and some tau fragments**. A) Scheme of tau and its fragments used in this analysis. B) Polymer formation after incubation of tau protein, N-terminal tau region, 3R tau fragment and 3RC tau fragment, with 3-APS. Inset shows the polymers found after incubation of tau peptide (residues 317–335) with 3-APS. Bars indicate 0.2 μm

Since 3-APS is not affecting to the microtubule network and to the binding of tau to microtubules, it is possible that it does not bind directly to the microtubule binding sites, found in tau molecule that are needed for tau-tau interaction [4]. To look for the binding site of 3-APS that results in tau aggregation we used different tau fragments (see Figure 6A). Figure 6B shows that tau aggregates were mainly found when tau 3RC peptide was mixed with 3-APS. Very few polymers were found when tau 3R peptide was tested and no polymers were observed for tau peptide containing the amino-terminal half region of tau protein. As can be seen also in the figure, larger polymers were found for the tau variant in which the N-terminal region was missing (tau 3RC) compared to the polymers found when the whole tau was assembled. These data suggest that 3-APS could bind to the C-terminal half region of tau protein, explaining the lack of interference of 3-APS addition with tau binding to microtubules, since tau may remain bound to microtubules and, also may bind to 3-APS through to a more distal C-terminal region. It should be indicated that tau binding to microtubules is mainly through the first and second tubulin binding repeats present in tau molecule [30].

It has been proposed that amyloid beta peptide fragment Aβ_1–28 _binds to 3-APS [19]. The sequence of this peptide being DAEFRHDSGY**EVHHQK**LVFFAEDVGSN K. The bold motif has been suggested to be the binding site for 3-APS [19]. Thus, we have looked for a similar motif in tau molecule and it has been found a related motif NIHHK in tau 3RC peptide. Thus, we have analyzed the polymerization of a tau peptide containing the residues 317–335, being NIHHK the residues 327 to 331 present in that peptide. Figure 6B (inset) shows that the indicated tau peptide (317–335) is able to assemble in the presence of 3-APS. This experiment suggests a posible direct interaction between tau peptide (residues 317–335) and 3-APS.

### The presence of 3-APS-tau aggregates is not toxic for cultured non neuronal cells

**Figure 7:**
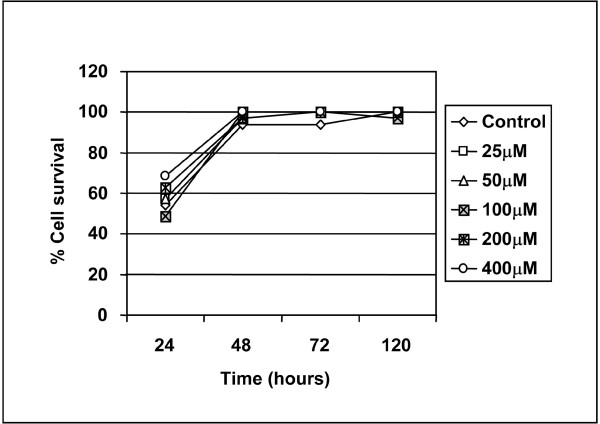
**3-APS treatment is not affecting cell survival**. Non neuronal (HEK 293 Tau cells) were treated with 3-APS. Viable and dead cells were stained with calcein AM and propidium iodide as described in "methods" and photographed at a fluorescence microscope. Percentages of specific cell survival were determined as described in "methods".

To HEK 293 cells stably transfected with tau, increasing amounts of 3-APS were added, and the cell viability was measured. The data from Figure 7 show that, after four days of 3-APS addition, the number of surviving cells was similar at the different 3-APS concentrations tested in the cell cultures and in controls without 3-APS. In every cell culture, the presence of cells containing tau aggregates was calculated based on Th-S staining, as previously shown in Figure 2D for HEK 293 cells expressing tau protein.

### Effect of 3-APS on neuronal cells

**Figure 8:**
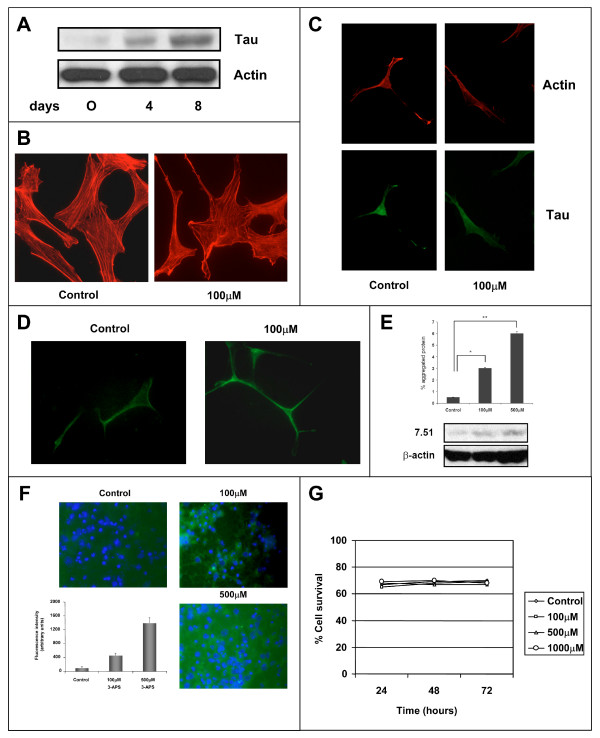
**The effect of 3-APS in neuronal cells**. A) Expression of tau protein was determined by western blot in non differentiated (0 days) and after starting differentiation for four days (4) or after eight days (8) with 2 mM dibutyril cyclic cAMP (cAMP) in SH-SY5Y neuroblastoma cells. Actin was taken as a control of loading protein. B) Effect of 3-APS on undifferentiated SH-SY5Y cells. In the absence (control) of 3-APS, or in the presence of 100 μM 3-APS. Cells were stained with phalloidin to label actin cytoskeleton. C) Effect of 3-APS on differentiated SH-SY5Y cells. SH-SY5Y cells differentiated for 8 days were untreated (control) or treated with 100 μM 3-APS. The cells were stained with antibodies raised against tau protein and with phalloidin to label polymerized actin. D) In the presence of 3-APS there is an increase, in differentiated SH-SY5Y cells, in Th-S staining. Untreated, or cells treated with 100 μM 3-APS were stained with Th-S and visualized by an immunofluorescence microscopy (see methods). An increase in Th-S staining upon 3-APS treatment was found. E) Detergent insoluble tau aggregates were isolated from neuroblastoma cells untreated or treated with 100 μM and 500 μM 3-APS. The amount of tau in detergent insoluble material was measured by testing its reaction with tau antibody 7.51. Actin was used as a loading control (see methods). F) Th-S staining in hippocampal neurons treated with 3-APS. Primary cultures of hippocampal neurons were stained with Th-S in the absence (control) or the presence of 100μM 3-APS or 500 μM 3-APS. The quantitation of the fluorescent intensity is shown. (G) Effect of 3-APS in hippocampal neurons viability. Primary hippocampal cultures were incubated in the absence (control) or the presence of 100 μM, 500 μM and 1000 μM, for 24, 48 or 72 hours and the percentage of cell survival was determined.

After looking at the effect of 3-APS in tau-expressing non neuronal cells, we have tested the effect of 3-APS in human SH-SY5Y neuroblastoma cells. These neuroblastoma cells express a very little amount of tau protein in undifferentiated cells state. However, upon differentiation (see methods), a high increase of tau protein was found in those cells (Figure 8A). When undifferentiated neuroblastoma cells were treated with 3-APS, no differences in phalloidin staining (actin cytoskeleton) were found (Figure 8B). In fact, stress fibers were observed (probably due to the low expression of tau protein). In addition, a very weak Th-S staining was observed in the absence or presence of 3-APS. Upon differentiation of SH-SY5Y cells with dibutyril cyclic AMP, stress fibers disappear (Figure 8C) and very few actin-tau membrane complexes, compared to those found in HEK 293 cells stably transfected with tau, were observed. When these differentiated neuroblastoma cells were treated with 3-APS none of those actin-tau complexes were found (Figure 8C). Moreover, in 3-APS treated cells an increase in Th-S staining was observed (Figure 8D). Also, an increase in detergent insoluble tau aggregates was found upon treatment of the differentiated SH-SY5Y cells with 3-APS (Figure 8E).

In addition, the effect of 3-APS on primary cultures of mouse hipoccampal neurons was analyzed. Figure 8F shows and increase in Th-S staining in 3-APS treated neurons. The effect of 3-APS addition in neurons viability was also studied, but not changes in cell viability was found in the absence or presence of 3-APS (Figure 8G). Finally, no differences in morphology of hipocacmpal neurons were observed, upon 3-APS addition.

## Discussion

The presence of amyloid deposits and tau aggregates are the main characteristics of Alzheimer's disease, and could be related to the neurodegenerative process. Usually, amyloid and tau pathologies are analyzed in an independent way, and is not known if drugs preventing amyloid pathology may also affect tau pathology. One example is 3-amino-1-propane sulfonic acid (3-APS), that maintains beta amyloid peptide in non fibrillary form [19], whose possible action on tau protein was unknown.

The aggregation of amyloid peptide, or tau protein, can be induced by sGAG, like heparin. However, in the case of amyloid peptide, low molecular weight heparin may reduce the accumulation of beta amyloid aggregates in a mouse model [17] and compounds, like 3-APS, that mimic the binding of sGAG, can bind to soluble non-fibrillar amyloid peptide, preventing amyloid aggregation [21]. It has been also suggested that the sulfate moieties of sGAG are sufficient to favour the formation of amyloid fibrils [15], 3-APS has a sulfate moiety, but it appears that this is not enough to induce amyloid aggregation.

In this work, we have found that 3-APS favours tau polymerization into fibrillar aggregates. The appearance of these tau aggregates was not toxic in our cell model, and the addition of 3-APS did not either result in toxicity to cultured neurons. A possible explanation is that 3-APS binds to tau protein through its C-terminal half region and, in this way, it is not affecting to the microtubule binding region present in tau protein, and therefore is not affecting to the microtubule binding function of tau. 3-APS has both a positively and a negatively charged regions. The positively charged region of 3-APS could bind to the negatively charged regions of tau protein, like those present at the C-terminal region of tau, and in consequence, the negative influence of the C-terminal region of tau on its selfassembly, would decrease upon 3-APS binding, allowing tau poymerization [31]. On the other hand the negative charge region of 3-APS could bind to the positive charges residues present in tau, in the C-terminal half of the molecule, residues that are involved in the binding of tau to actin. If it the case 3-APS and actin should compete for the same tau-binding site. Also, it has been indicated that tau binding to actin is through a region closed to the C-terminal of tau molecule [29]. This region contains the NIHHK motif, similar to the VHHQK motif that it has been suggested to be required for the binding of 3-APS to Aβ peptide [19].

Thus, in the presence of 3-APS, the binding of tau to actin could be decreased if 3-APS overlaps its tau binding site with that of actin. We can not exclude that 3-APS may also bind to the negative charged N-terminal region of tau, with a similar effect on tau assembly [32], since we did not test a tau variant containing the N-terminal and the tubulin binding regions. Indeed, that possible binding of 3-APS to the N-terminal region of tau may influence the binding of tau to membrane-actin aggregates [33].

The proposed mechanism to explain the assembly of tau in the presence of 3-APS could not be very different to that suggested for heparin-induced tau assembly. In both cases a tau peptide comprising residues 317 to 335 is enough to form aggregates [34]. In addition, 3-APS could act as a sequestering molecule for tau, but it could not be essential for cell viability. In this way it has been described that the mice lacking tau could develop and live like their wild type counterparts [35].

There is evidence for a role of sGAG in the formation of amyloid and tau aggregates [36] and, as indicated in the introduction, a possible toxic effect by promoting amyloid peptide aggregation was suggested for sGAG, but, conversely, it has been described that sGAG like heparan or chondroitin sulfate attenuate the neurotoxic effect of amyloid peptide in primary neuronal cultures [37], and a role for heparan sulfate as a modulator of Aβ formation, through beta secretase, has been suggested [38]. It will be of interest in further studies, to test for a possible effect of 3-APS on beta secretase activity.

Regarding tau protein, it has been reported that the chondroitin sulfate content inversely correlates with the amount of hyperphosphorylated tau in cortical areas of Alzheimer's disease patients [39]. Thus, a posible neuroprotective role of sGAG in Alzheimer's disease could not be excluded, and the neuroprotective role could be extended to some related compounds like 3-APS, because 3-APS could be used for decreasing Aβ pathology and, although it aggregates tau protein, it is not toxic for cultured cells. Moreover, 3-APS promotes the decrease of tau-actin complexes [28,29] that could be toxic for the cells [24].

## Conclusion

We suggest that drugs of potential interest for the treatment of Alzheimer's disease should be tested not only on one of the proteins involved in Alzheimer's disease pathology, like amyloid peptide, but also on the component of the other pathological hallmark of Alzheimer's disease, tau protein. Our data, although support the lack of effect of 3-APS in cell viability, indicate that 3-APS could promote tau aggregation, probably reducing the amount of available functional tau inside of a cell. This relevant aspect should be taken into account for a possible future use of Tramiprosate in human beings.

## Methods

### Materials

3-Amino-1-propanesulfonic acid (3-APS) (Ref. A4147), Phenylmethylsulfonyl fluoride (PMSF), EDTA, EGTA, 2-Mercaptoethanol, MES, HEPES, Trizma Base, NaF, Na_3_VO_4 _and SDS were purchased from Sigma. Triton X-100, Tween-20, NaCl and MgCl_2 _were obtained from Merck. Acrylamide/bisacrylamide solution and Bradford reagent was supplied by Bio-Rad. Protran Nitrocellulose Transfer Membrane was from PerkinElmer. The chemiluminiscent detection kit (Western Light) was purchased from Tropix. DAPI (Ref. 268298) was obtained from Calbiochem. Thioflavin S (Th-S) (Ref. T-1892) was purchased from Sigma.

### Antibodies

For immunoblot analysis, we used anti-β-tubulin (1/2000; Sigma), anti-β-actin (1/2500; Sigma), and 7.51(anti-tau antibody; a gift from Dr. C. M. Wischik, UK) antibodies followed by relevant secondary antibodies (1/2000; DAKO).

For immunofluorescence, we used anti-β-tubulin (1/500; Sigma), Tau-5 (1/500; Chemicon) and T14(1/500; Zymed Laboratories). The secondary antibodies (Molecular Probes) were used at 1/1000.

Actin staining was done by using phalloidin TRITC (1/300; Sigma). 0,01% Thioflavin S in PBS 1× was used for staining of treated or untreated cell cultures.

### Reverse transcription of RNA and polymerase chain reaction (RT-PCR)

Total RNA from HEK 293 and HEK 293 tau expressing cells were prepared using the reagent TRIzol (Invitrogen) and following the supplier's protocol. Reverse transcription was performed using the first cDNA synthesis kit (Roche Applied Science) on 5 μg of RNA with oligo(dT) primers. PCR was performed with the primers for tau R1 (5'-GGCGAATTCGGATCCATGCCAGACCTGAAGAATG-3') and R2 (5'-GGCCTGC AGTTACTCGCGGAAGGTCAGCTTGTGGG-3'). The amplifications were performed basically with the following protocol: 30 cycles of 94°C for 45s, 55°C for 1 min, and 72°C for 1 min. The PCR products were resolved on a 1% agarose gel and stained with ethidium bromide. As a loading control, RT-PCR for actin was performed for each sample. The used primers for murine actin were H1 (5'-GCATGGAGTCCTG TGGCATCCACG-3') and H2 (5'-GGGTGTAACGCAACTAAGTCATAG-3'). Differences among groups were analysed by Student's unpaired *t*-test to determine significant differences between means.

### Cell culture

#### HEK 293 tau cells

HEK 293 tau expressing cells (expressing tau 3R isoform, a kind gift from Dr. Miguel Medina) were grown in Dulbecco's modified Eagle's medium (DMEM) supplemented with 10% fetal bovine serum, 2 mM Glutamine, 1 mM Piruvate, 100 U/ml penicillin, 100 U/mL streptomycin, and 0.2 mg/ml Zeocin in a humified atmosphere of 5% CO_2_/95% air at 37°C. Proliferating HEK 293 tau expressing cells were plated on glass cover slips coated with 1 mg/ml poly-L-lysine, maintained in the same culture medium during 24 hours and treated with 3-APS for 24,48 and 72 hours , at 100 μM, 500 μM 3-APS.

#### SH-SY5Y cells

Human neuroblastoma SH-SY5Y cells [40] were maintained in Dulbecco's modified Eagle's medium (DMEM) supplemented with 10% fetal bovine serum, 2 mM Glutamine, 100 U/ml penicillin, 100 U/mL streptomycin in a humified atmosphere of 5% CO_2_/95% air at 37°C. For differentiation, proliferating SH-SY5Y cells were plated and then cultured in Neurobasal-B27 medium (Gibco, Grand Island, NY) supplemented with 2 mM dibutyril cyclic AMP and 1 mM glutamine for 7 days. At this time, about 90% of the cells has extended long neurites and became postmitotic (showing no significant incorporation of tritiated thymidine into DNA).

After 7 days differentiated SH-SY5Y cells were treated during 48 hours with 100 μM, 500 μM 3-APS.

#### Primary culture

Hippocampal neurons were cultured as described by Banker and Cowan [41]. Pregnant wild type females were sacrificed at gestional day 18, and the embryos were removed in sterility. Dissociated hippocampal neurons were plated on glass cover slips coated with 1 mg/ml poly-L-lysine and then 20 μg/ml laminin. After incubating in medium containing 10% horse serum (Gibco-BRL, Gaithersburg, MD) for 24 hours, the medium was changed and supplemented with N2 and B27 (Gibco-BRL). At this point, neurons were treated with 3-APS for 24, 48 and 72 hours, at 100 μM, 500 μM 3-APS.

### Cell Lysis and Western Blot Analysis

HEK 293 and HEK 293 tau expressing cells were washed once with phosphate-buffered saline (PBS), placed on ice, and then homogenized in a buffer containing: 20 mM HEPES, pH 7.4; 100 mM sodium chloride (NaCl); 100 mM sodium fluoride (NaF); 1% Triton X-100; 1 mM sodium orthovanadate (Na_3_VO_4_); 5 mM EDTA; and the Complete protease inhibitor cocktail (Roche Diagnostics, Barcelona, Spain). After determination of the protein content via Bradford assay, samples containing the same amount of protein were mixed with electrophoresis buffer containing sodium dodecyl sulfate (SDS), boiled for 5 min, and separated by gel electrophoresis in the presence of SDS on 10% acrylamide gels. The proteins were then transferred to nitrocellulose membranes by following standard procedures, and the membranes were blocked with 10% nonfat dried milk in PBS, 0.2% Tween-20 (PBST). The blocked membranes were incubated overnight with primary antibodies diluted in blocking solution at 4°C. The membranes were then rinsed three times in PBST and incubated with the corresponding peroxidase-conjugated secondary antibody for 1 hr at room temperature. The immunoreactive proteins were visualized by using an enhanced chemiluminescence detection system (Amersham), and subsequent densitometric analysis was performed with an imaging densitometer (GS-710 model; Bio-Rad, Hercules, CA). Western blot analysis were also done using an antibody raised aginst actin (Sigma) as loading control. Protein extracts prepared from differetiated SH-SY5Y cells were processed and analysed by Western Blot using tau antibody 7.51 (1/100) as previously indicated.

### Immunoblot analysis of aggregated Tau

Treated HEK 293 tau expressing cells, or SH-SY5Y neuroblastoma cells, were homogenized in RIPA buffer (50 mM Tris-HCl (pH 8.0), 150 mM NaCl, 5 mM EDTA, 1% Nonidet P-40, 0.25% sodium deoxycholate, 1% SDS, 5 mM 4-(2-aminoethyl) benzene-sulfonyl fluoride hydrochloride, 1 μg/ml proteases inhibitors (Complete protease inhibitor cocktail (Roche Diagnostics, Barcelona, Spain)), 1 mM NaF, 1 mM Na_3_VO_4_, and 1 mM β-glycerophosphate) without thawing by using a polytron homogenizer (Kinematica, Kriens, Switzerland) at its highest speed for 30 s. Before centrifugation to fractionate the detergent soluble from detergent insoluble material, an aliquot from each sample was taken to determine if each sample contains, or not, a similar amount of actin (loading control). To do that we have used an antibody raised against actin. Tau protein aggregates from homogenates were isolated by centrifugation (4°C) for 20 min at 20000–30000 g (table-top centrifuge at 15000 rpm). One fourth of the supernatant volume, after centrifugation, was taken to characterize the protein present in that fraction. The whole aggregated protein present in the insoluble fraction was then diluted in O+ buffer (62.5 mM Tris-HCl pH 7.0, 10% (w/v) glycerol, 5% (v/v) β-mercaptoethanol, 2.3 % (w/v) SDS, 1 mM EDTA, 1 mM EGTA, 1 mM NaF, 1 mM Na_3_VO_4 _1 mM PMSF (phenylmethyl-sulfonyl fluoride), 1 μg/ml proteases inhibitors (Complete protease inhibitor cocktail (Roche Diagnostics, Barcelona, Spain)), boiled for 5 min, and separated by gel electrophoresis in the presence of SDS on 10% acrylamide gels. Then, the protein content was analized by western blot as previously described.

### Immunofluorescence analysis

After treatments, HEK 293 and HEK 293 tau expressing cells were fixed with either cooled methanol (-20°C) (tubulin immunofluorescence) or 4% paraformaldehyde (actin and tau immunofluorescence) for 20 min at 4°C or 37°C, respectively, and then washed with buffer A (0.1 M MES; 2 mM EGTA; 0.5 mM MgCl_2_) or with phosphate buffered saline (PBS), respectively. Fixed cells were incubated with 1 M glycine 30 min then permeabilized with 0.2% Triton X-100 in PBS or buffer A for 5 min at room temperature. The cover slips were blocked with 1% BSA/PBS or Buffer A for 1 h at room temperature and incubated in primary antibodies in 1% BSA, in PBS, or in buffer A, for 1 h at room temperature. After washing three times with PBS or with buffer A, the secondary antibodies were incubated for 1 h, at room temperature. DAPI (1 μg/ml) staining was performed 10 minutes before finishing secondary antibody incubation. Finally, the covers lips were washed three times with PBS or with buffer A and once with H_2_O, and mounted with FluorSave Reagent (Calbiochem).

Fluorescence microscopy was used to measure fluorescence intensity. After staining with 0.01 % Th-S and T14 antibody cell were observed on a Zeiss Axiovert200 fluorescent microscope. T14 antibody and thioflavin-S fluorescence images were captured through a 100× objetive on a high resolution CCD camera (SPOT RT Slider, Diagnostic). The images were saved for later analysis and quantitation. Camera exposure and light settings were keot constant during each experiment. The fluorescence intensity measurements were carried out using the image analysis software Metamorph 6.1 r6 (Universal Imaging).

A similar protocol was followed for SH-SY5Y neuroblastoma cells and primary cultures of hippocampal neurons.

### Binding of tau to microtubules

For *in vitro *assembly of tau protein with microtubules, purified tubulin was assembled in the presence of taxol [42] and mixed with tubulin, after incubation of the mixture, the polymerized protein was isolated by centrifugation in Airfuge (Beckman) at room temperature, for 15 min at 100000 g. The protein present in supernatant and pellet was analyzed by gel electrophoresis followed by western blot using anti-tau and anti-tubulin antibodies.

### Determination of Cell Viability

Cell viability was assessed by calcein-propidium iodide uptake [43]. Calcein/acetoxymethyl ester is taken up and cleaved by esterases present in living cells, yielding yellowish-green fluorescence. In contrast, propidium iodide is taken up only by dead cells, which then exhibit orange-red fluorescence. Briefly, cells were incubated for 30 min with 8 μM propidium iodide (Sigma) and 1 μM calcein/acetoxymethyl ester (Molecular Probes). The cultures were then rinsed once with Hanks balanced salt solution containing 2 mM CaCl_2_, and the cells were visualized by fluorescence microscopy with a Zeiss Axiovert 135 microscope. Three randomly selected fields were analyzed per well (100–200 cells/field) in at least three independent experiments. Cell viability was expressed as the percentage of calcein-positive cells with respect to the total number of cells.

### Protein and peptide preparation

Recombinant human tau (whole molecule), N-terminal tau (residues 1–251), peptide 3RC (containing three tubulin binding motifs and the carboxyl-terminal region), peptide 3R (containing three tubulin binding motifs), have been synthesized and purified as previously reported [4]. Tau peptide containing residues 317–335 was obtained as previously indicated (meter referencia 33).

### Assembly of Tau Peptides into Filaments

Filaments were grown by vapor diffusion in hanging drops in the standard way used for protein crystallization as previously indicated [44]. In a typical experiment, 0.5–2 μg of peptide was resuspended in 10–15 μL of buffer A (0.1 M MES (pH 6.4), 0.5 mM MgCl_2_, and 2 mM EGTA) containing 50 mM NaCl and 3-APS at concentrations ranging from 1 to 4 mM. In other assays, a similar amount of tau (0.5–2 μg) but in the presence of 0.5 mg/mL heparin was resuspended in buffer A [4]. The reservoir in this case contained 0.2 M NaCl in buffer A. Filaments were obtained after incubation for 4 days at 4°C. The samples we revisualized by electron microscopy as described [4]. Electron micrographs were obtained at a magnification of 80000 on Kodak SO-163 film. Micrographs were digitized using an Eikonix IEEE-488 camera with a pixel size equivalent to 7 Å in the specimen plane. Processing and measurements were performed using the Digital micrograph 2.1 software from Gatan. Several standards were used for the control of the measurements. Alternatively, tau polymers were stained with Th-S and visualized by immunofluorescence [27].

## Abbreviations

Aβ : amyloid-β-peptide; sGAG: sulfated glycosaminoglycans; 3-APS: 3-amino-1-propanesulfonic acid; Th-S: Thioflavin S.

## Competing interests

The author(s) declare that they have no competing interests.

## Authors' contributions

IS did the main experimental work, helped and with the advice of FH and FJM. JDR advised in some aspects of the work. JA is responsible in designing and overseeing the experiments and, together with IS, wrote the manuscript. All authors have read and approved the final manuscript.

## Supplementary Material

Additional file 1 **Tau binding to microtubules in the presence of 3-APS**. Tubulin (2 μM) was incubated in the presence of 10 mM Taxol for 30 min at 37°C; and, afterwards; 0.2 μM tau and increasing amounts of 3-APS were added to different aliquots. After 10 min of incubation at 37°C, the polymerized and unpolymerized protein fractions were isolated by centrifugation and the amount of tau protein and tubulin in the unpolymerized (S) and polymerized (P) protein was determined by western blot, using antibodies against those proteins. The percentage of tau protein present in the polymerized protein, at different 3-APS concentrations, respect to that found in the absence of 3-APS, was determined. Error bars, from three different experiments, are shown. Click here for file
