# Supplementary figures and images for: Tramiprosate, a drug of potential interest for the treatment of Alzheimer's disease, promotes an abnormal aggregation of tau
Source: Mol Neurodegener. 2007 Sep 6;2:17. doi: 10.1186/1750-1326-2-17 (PMC2048960; doi:10.1186/1750-1326-2-17)

**A**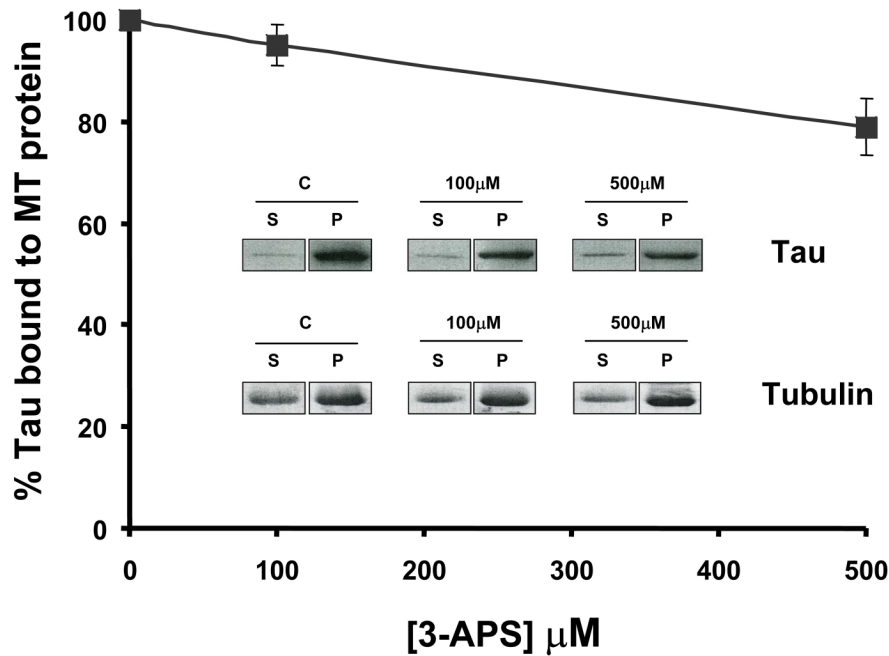

Supplement: Additional file 1 — Tau binding to microtubules in the presence of 3-APS. Tubulin (2 μM) was incubated in the presence of 10 mM Taxol for 30 min at 37°C; and, afterwards; 0.2 μM tau and increasing amounts of 3-APS were added to different aliquots. After 10 min of incubation at 37°C, the polymerized and unpolymerized protein fractions were isolated by centrifugation and the amount of tau protein and tubulin in the unpolymerized (S) and polymerized (P) protein was determined by western blot, using antibodies against those proteins. The percentage of tau protein present in the polymerized protein, at different 3-APS concentrations, respect to that found in the absence of 3-APS, was determined. Error bars, from three different experiments, are shown. [file 1750-1326-2-17-S1.pdf]
